# Supplementary material for: Survival Motor Neuron Protein Participates in Mouse Germ Cell Development and Spermatogonium Maintenance
Source: Int J Mol Sci. 2020 Jan 25;21(3):794. doi: 10.3390/ijms21030794 (PMC7037566; doi:10.3390/ijms21030794)
Supplement: Supplementary file 1 [file ijms-21-00794-s001.pdf]

SUPPLEMENTAL INFORMATION

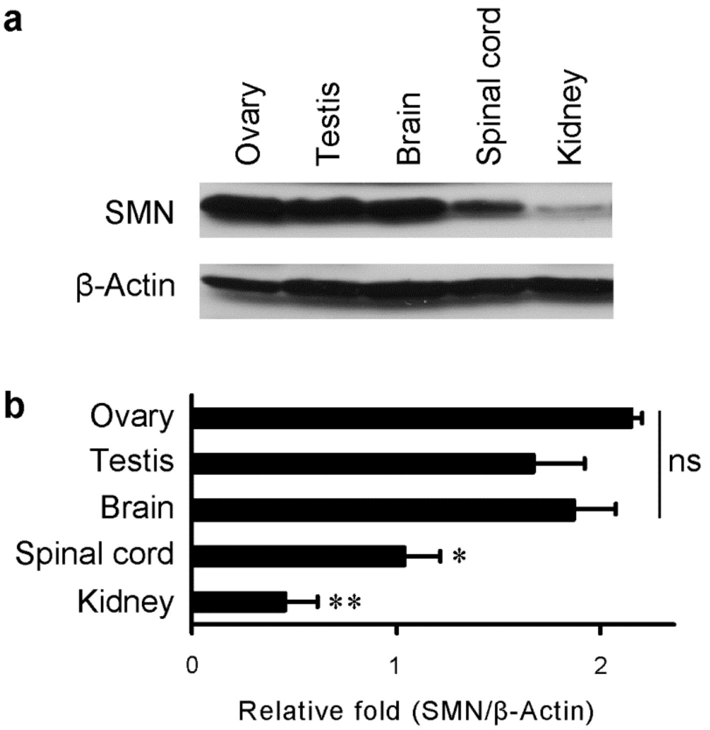

Supplementary Figure S1. SMN expression profiles in different adult mice tissues. (A) Western blot of SMN expression in testis, ovary, brain, spinal cord and kidney; β-Actin serves as the internal loading control (n = 3 mice per organ). (B) Quantification of SMN expression in adult mice tissues. SMN expression is normalized to the β-Actin expression level. SMN protein shows high level in the ovary, testis and brain, and slightly low level in the spinal cord and kidney. *, indicates significance (p<0.05); ns, indicates no significance.

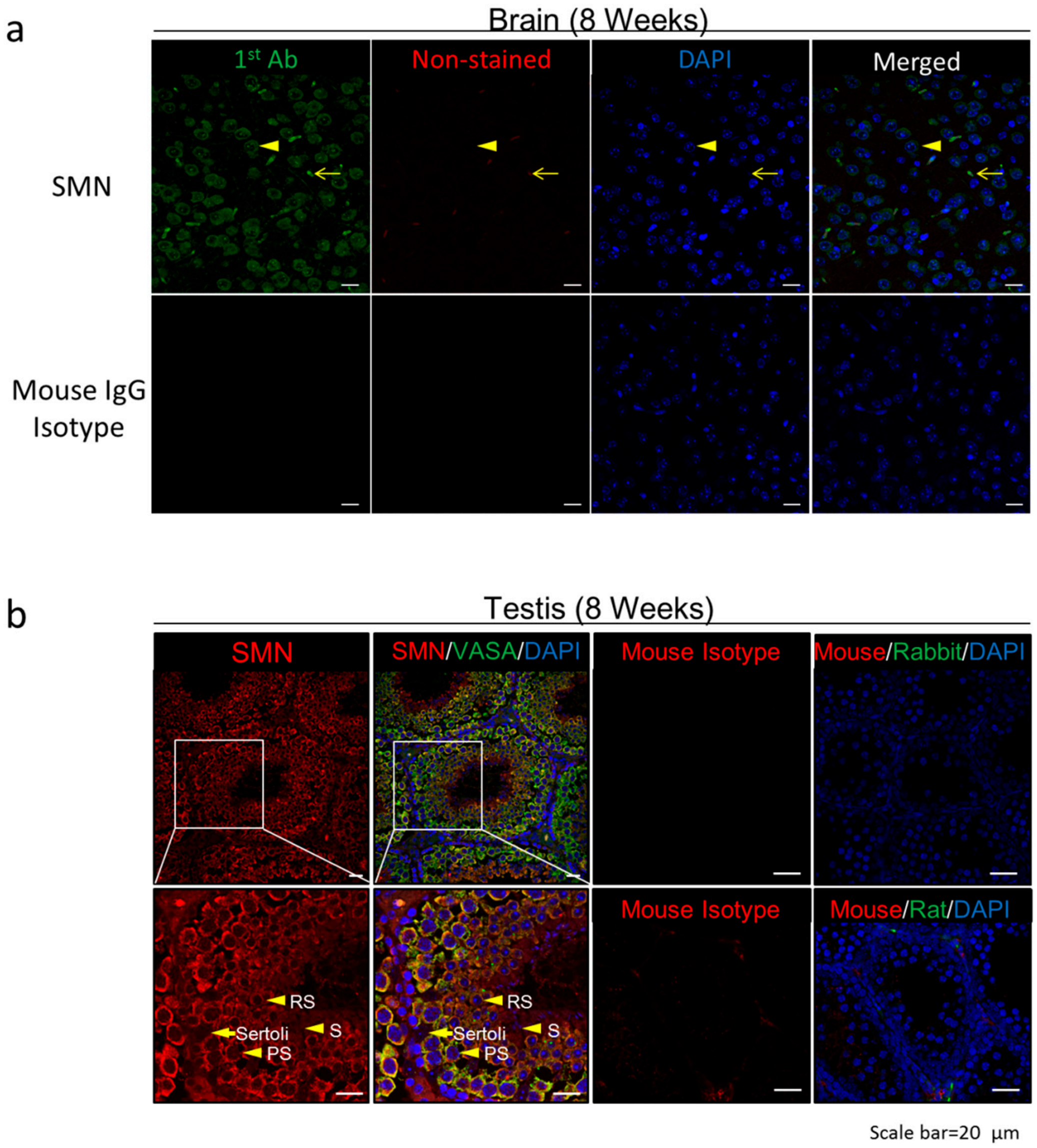

Supplementary Figure S2. Specificity of SMN antibody confirmed in mouse brain section and testis. (A) In 8 weeks old mouse brain, SMN protein is detectable by mouse anti-SMN antibody. For negative control, mouse isotype IgG and rat isotype IgG 2nd antibodies conjugated with 488 fluorescent dye is used. There is many SMN positive cells in the brain section (green color, arrow head), and some autofluorescence that can be observed in intracellular region in both 488 and 546 channels (green and red color, arrow indicated). (B) In the testes from 8 weeks old adult mice, cells doubly stained with SMN (red) and germ cell-specific markers,

VASA, show colocalization in lower and larger magnification (arrowhead) by IHC staining. Sertoli cells (arrow indicated) expresses background level of SMN. RS: round spermatid. S: sperm. PS: pachytene stage spermatocytes.

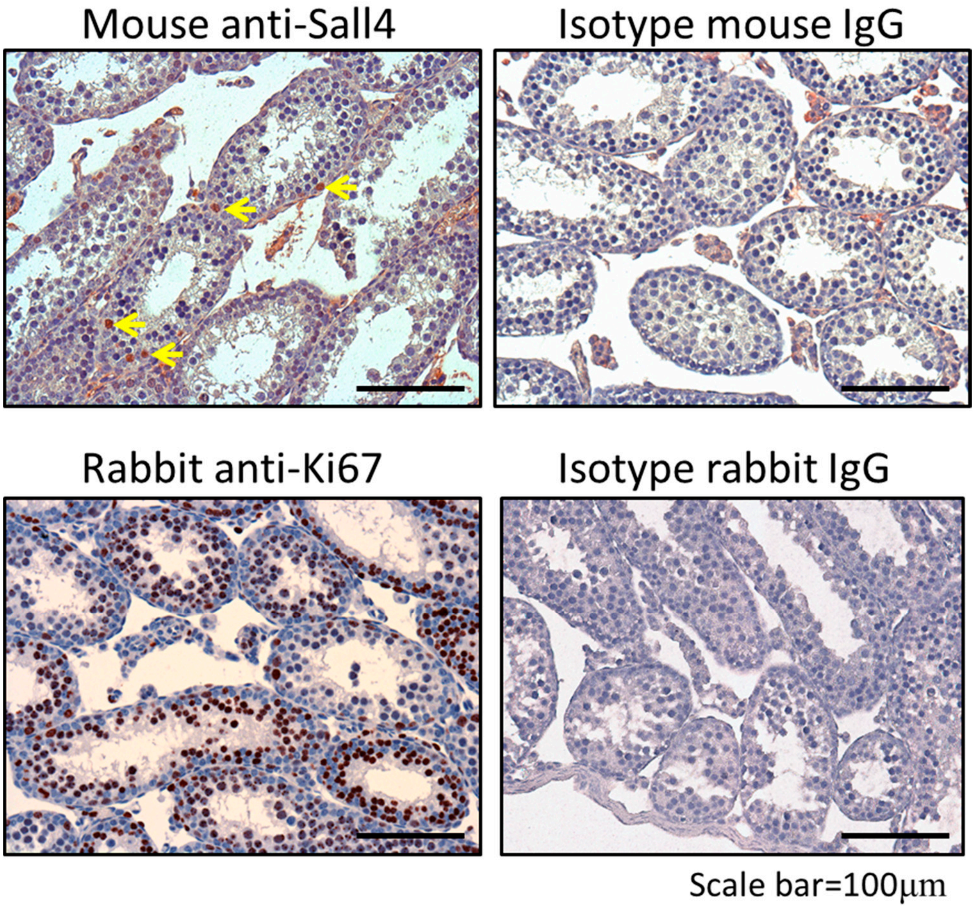

Supplementary Figure S3. IHC staining control in mouse testis section. Mouse anti-Sall4 antibody (H00057167-M03, Novus Biologicals) is used to evaluate the background signal of mouse testis section when using mouse antibody. The isotype mouse IgG control showed no signals inside the seminiferous tubules, but only some background in the intratubule regions (right panel). Rabbit IgG control group also detects no background (right panel). Sall4 antibody specifically is stained in the nuclei of type A_{single}-like spermatogonia (left panel, arrow indicated) and MKi67 antibody also shows positive signals in the nuclei (left panel), indicating the successful staining results.

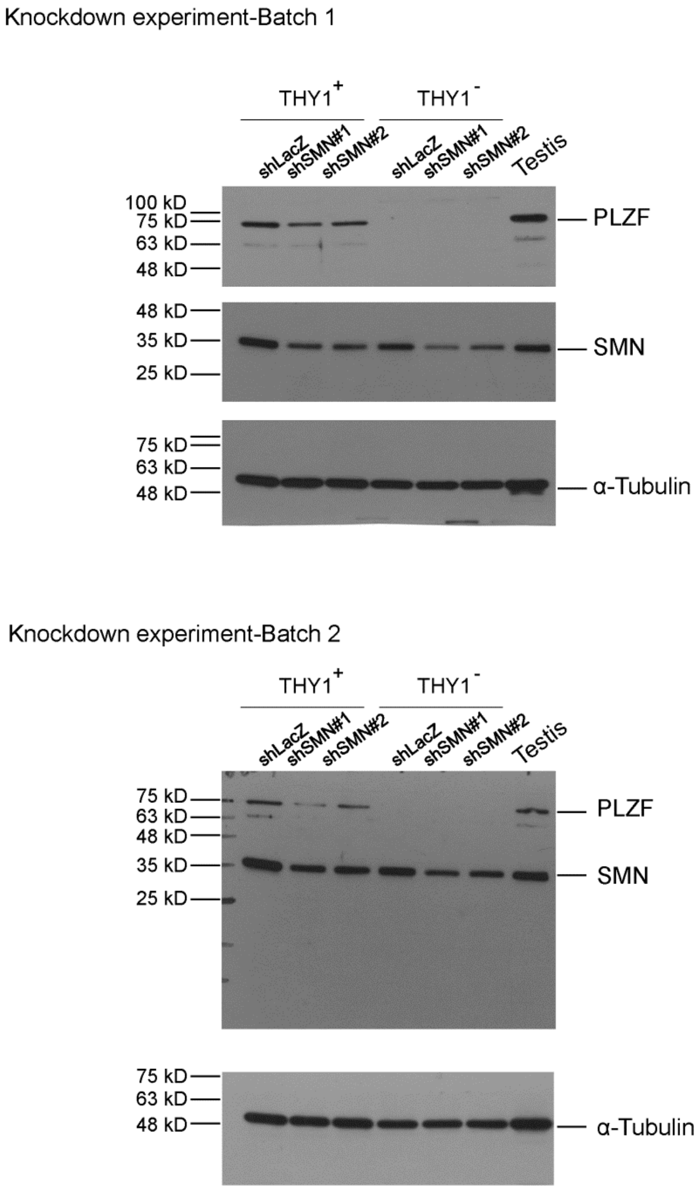

Supplementary Figure S4. Original western blots for main figures. Complete western blots of PLZF, α-Tubulin and SMN expression in SSCs after SMN knockdown shown in Figure 3A for two batches of experiments. The molecular weight standards are shown on the left. Whole testis is used as the positive control for each protein.

Supplementary Table S1. Sequences of primers used for genotyping of SMA transgenic mice (related to Figure 2) and real-time PCR analysis (related to Figure 3).

Primer sets used for SMA mice genotyping

For Smn1	SEQUENCE
3' S1	ATAACACCACCACTCTTACTC
5' S2	GTAGCCGTGATGCCATTGTCA
5' H1	AGCCTGAAGAACGAGATCAGC
For human SMN2	SEQUENCE
2F	CGAATCACTTGAGGGCAGGAGTTTG
2B	AACTGGTGGACATGGCTGTTCATTG

Primer sets used for real-time PCR analysis

TARGET	FORWARD SEQUENCE	REVERSE SEQUENCE
Smn1	GCTCCGTGGACCTCATTTC	GGGCCGTTGAATTTTAGACC
Plzf	GCATTTACTGGCTCATTCAGCG	GTGCGCTTTGTGCCTGAAA
Sall4	AATGCTGTGCCGAGTTCTTT	GTGCCCAGCTTCTTCAAGTC
Acr1	CCAGGTTAGGGCAGGAGTATG	TAACCCACAGGGACCATCACA
Scp3	TCAGATGCTTCGAGGGTGTG	CTGGAGCCTTTTCATCAGCAAC
Trp53	GCAACTATGGCTTCCACCTG	TTATTGAGGGGAGGAGAGTACG
Puma	ACCGCTCCACCTGCCGTCAC	ACGGGCGACTCTAAGTGCTGC
Bax	GTGAGCGGCTGCTTGTCT	GGTCCCGAAGTAGGAGAGGA
Gapdh	TGGCCTTCCGTGTTCCTA C	GAGTTGCTGTTGAAGTCGCA
